# Circumventing cigarette regulation: Product characteristics of cigarette-like cigarillos on the Dutch market

**DOI:** 10.18332/tid/167476

**Published:** 2023-07-14

**Authors:** Jeroen L. A. Pennings, Charlotte G. G. M. Pauwels, Erna Schenk, Reinskje Talhout

**Affiliations:** 1 Centre for Health Protection, National Institute for Public Health and the Environment (RIVM), Bilthoven, The Netherlands

**Keywords:** cigarillos, product regulation, EU-CEG

## Abstract

**INTRODUCTION:**

Cigarillos have been reported to provide an alternative to cigarettes with a characterizing flavor, which are banned in the European Union. Additionally, they are cheaper than cigarettes. To better inform policy making, we wanted to gain better insight into the market situation for cigarillos in the Netherlands.

**METHODS:**

We analyzed product data notified by manufacturers via the European Common Entry Gate system (EU-CEG), as extracted on the 1 June 2022. First, we identified parameters that allowed classifying cigarillos into cigar-like, cigarette-like and intermediate-type cigarillos. Next, we compared product characteristics for these groups.

**RESULTS:**

We identified five parameters that allowed classifying cigarillos into subtypes: product weight, filter presence, leaf tobacco percentage in the product, flue-cured tobacco percentage in the product, and the number of flavorings. Almost a quarter (71/285) of the cigarillos on the Dutch market were classified as cigarette-like. Compared to other cigarillo types, these have a high number of flavorings (average n=43), including many sweet and fruity flavorings. The package types of cigarette-like cigarillos resemble those of cigarettes. However, 85% of the cigarette-like cigarillos are available in smaller pack sizes than allowed for cigarettes. When comparing data over the period 2019–2022, we found a decrease in the number of cigar-like cigarillos and an increase in the number of cigarette-like cigarillos, which hints at a shift in the market composition.

**CONCLUSIONS:**

Cigarette-like cigarillos can provide a way to evade cigarette regulation. Moreover, their characteristics make them attractive for consumers, including young people. Regulators should consider amending regulations to close the regulatory loopholes that allow evading tobacco legislation.

## INTRODUCTION

Cigarillos are commonly considered as small or medium-sized cigars and in some countries they can also be referred to as ‘little cigars’. Indeed, within the context of European Union (EU) legislation, cigarillos are defined as a small type of cigar of a maximum weight of 3 g each^1,2^, with the difference between cigarettes versus both cigars and cigarillos defined primarily based on their outer wrapping^3^, definitions that we will use in this study. However, some cigarillos resemble cigarettes in size and shape, are equipped with a filter, and/or have characterizing (i.e. a clearly noticeable non-tobacco)^1^ sweet flavors, such as fruit or candy, that appeal to adolescents and young adults^4,5^. In addition to characterizing flavors, they have lower taxation^6,7^ and fewer units per pack, which makes them affordable and appealing to consumers, including young people^8-10^. Their appeal to young people is even more concerning given their promotion on social media such as TikTok and Instagram^11,12^.

A study by Havermans et al.^13^ found that in 2020, current cigarillo use among Dutch adults was 2%, which was considerably lower than the 19% for current cigarette use. However, ever use of cigarillos was 13%, which was higher than in previous years or values reported for most other European countries. Cigarillo use was highest among participants who indicated a preference for menthol flavored cigarettes. Also, cigarillo users indicated that the availability of flavors is an important reason for their using cigarillos. This might indicate a shift from people smoking menthol flavored cigarettes to smoking menthol flavored cigarillos after the introduction of the ban on characterizing menthol flavors in cigarettes and roll-your-own tobacco^13^. It can be noted here that Article 7.12 of the Tobacco Products Directive (TPD) exempts cigarillos from the ban on tobacco products with a characterizing flavor^1^.

The combination of product characteristics and legislation make cigarillos suitable as an alternative to cigarettes that circumvents regulation on (fruit or menthol) flavors, pack size and taxation. Indeed, cigarillos that bypass such regulations are available in, among others, the UK and USA^7,14^. Nevertheless, studies on additive use indicated that there is also considerable diversity among cigarillos in the Netherlands, ranging from cigar-like to flavored cigarette-like products^15^. Further insights into cigarillo diversity and characteristics in the Netherlands would be valuable from a regulatory point of view to determine if and how regulations would need revising, for example the forthcoming revision of the EU Tobacco Products Directive, as well as tobacco legislation outside the EU.

To this end, we determined for the Dutch market to what extent distinct cigarillo subtypes can be distinguished based on variation in product characteristics; how such subtypes compare to cigars and cigarettes; if the market supply for product subtypes changes over time; and to what degree cigarillos, or cigarillo subtypes, can be seen as a way to circumvent regulation.

## METHODS

### Data

Data for this study were obtained from the European Common Entry Gate system (EU-CEG)^16^. This is a database in which manufacturers and importers are legally obliged to provide information about the composition and other properties of the tobacco and related products they market in each European Union Member State. EU-CEG therefore provides a valuable source of information to national competent authorities for tobacco or e-cigarette data analyses^17,18^. We used data as available for active products in the Dutch section of the EU-CEG system on 1 June 2022. Additionally, to determine changes over time in cigarillo characteristics, cigarillo data were extracted for every 3-month period from June 2019 to December 2022. Product data were exported and further statistical analyses were carried out in R (V.4.2.0) and Microsoft Excel.

In total, 72 parameters were available for analysis, based on their applicability to cigarillos as well as mandatory notification in EU-CEG. These involved general parameters such as product weight, length, or filter presence (n=7); tobacco composition such as cure method (n=19); and additive use such as total number of additives, number of additives per function of product part, total additive weight, and total flavoring weight (n=46).

### Cigarillo subtype classification

Initial multivariate data visualization was done using Principal Component Analysis (PCA)^19^. PCA is a mathematical algorithm that reduces the complexity (dimensionality) of large data sets by visualizing data points in a smaller number of dimensions called principal components. Principal components are mathematically derived as combinations of parameters and this is done in such a way that principal components capture as much variation as possible in a low number of dimensions. For data visualized this way, the more similar two data points (here: cigarillo products) are in their characteristics, the more closely they will appear in the visualization. A less mathematical explanation of PCA can, for example, be found in Cheng^20^.

Next, for each of the parameters, we assessed by a density plot if their distribution could be considered as bimodal. Parameters for which visual evaluation indicated a strongly bimodal (as opposed to a weakly or non-bimodal) distribution were considered as potentially indicative for two groups. Additionally, we determined if each parameter allowed for discriminating between cigarettes and cigars. Parameters with a receiver operating characteristic (ROC) area under the curve (AUC) (two-tailed) greater than or equal to 0.9 were considered as significantly discriminating. This value is usually considered as giving excellent discrimination^21^. For parameters that met both criteria, we used mutual Spearman correlations and product knowledge to remove redundancy. As an example, the percentage Virginia tobacco is highly correlated to the percentage flue-cured tobacco as this is a common cure method for Virginia tobacco. In cases where redundancy was found, we kept the parameter deemed most informative.

For the set of parameters obtained by the previous steps, we applied logistic regression modelling to discriminate between cigarettes and cigars. Logistic regression yields a formula that gives a score ranging from 0 (fully cigar-like) to 1 (fully cigarette-like), based on whether the value for a parameter (e.g. weight) was more similar to a cigar (higher weight) or a cigarette (lower weight); this was done for each parameter separately. The resulting formulas were combined into a prediction model that gave the average of the individual parameter-based prediction values as output. This model was applied to cigarillo data to determine a logistic-regression-based product-type score between 0% (fully cigar-like) to 100% (fully cigarette-like). Cigarillos with a score ≤30% were assigned as being cigar-like, those with scores ≥70 % as cigarette-like, and the remainder as being of intermediate-type.

### Cigarillo subtype comparison

For the three cigarillos subtypes, as well as cigars and cigarettes, product characteristics were compared, regarding previously mentioned parameters as well as package type, average and smallest pack size available per product, and the most frequently used flavorings per group. For flavorings that were in the top 10 of most frequently used in either of these five product groups, their use prevalence in the various groups was visualized as a heatmap combined with hierarchical clustering (Euclidean distance, Ward’s linkage). Flavor descriptions were obtained from the Leffingwell database, which contains flavor data relevant to the food, beverage and tobacco industry^22^.

Finally, we determined the product-type scores for cigarillo data ranging from June 2019 to December 2022 to evaluate changes over time. To assess changes in the market, we compared the number of products per subtype as well as the percentage cigarette-like cigarillos over time, using the Spearman rank correlation with Spearman’s rho and the corresponding p-value as output, with p<0.05 (two-tailed) considered significant. It may be noted here that the last two time points were later than the time point used for the bulk of the analyses; this is because data for those later time points became available during the writing phase of the manuscript.

## RESULTS

### Data

On 1 June 2022, the number of distinct products (based on brand name, brand subtype name and product composition) that were registered in EU-CEG as active for the Dutch market were 489 for cigarettes, 3555 for cigars and 285 for cigarillos. For these products, 72 product parameters were available for analysis. An overview of these parameters is given in Supplementary file Table 1.

**Table 1:** Product group comparisons by number of products, key parameters, package type* and pack size

*aracteristics*	*Cigarette*	*Cigar*	*Cigar-like cigarillo*	*Intermediatetype cigarillo*	*Cigarette-like cigarillo*
**Number of products**	489	3555	140	74	71
**Key parameters**					
Product weight (mg), median (IQR)	838 (113)	14196 (7070)	1532 (900)	1303 (659)	998 (253)
Filter present (%)	98	0.1	0	30	92
Leaf tobacco (%), mean (SD)	63 (13)	100 (1)	99 (5)	88 (17)	82 (13)
Flue-cured tobacco (%), mean (SD)	61 (17)	0 (3)	0 (1)	10 (19)	43 (23)
Number of flavorings, mean (SD)	16 (15)	0 (3)	1 (6)	19 (31)	43 (44)
**Package type** (%)					
Flip-top box, square corner	86	5	1	8	13
Shoulder hinged box	1	3	14	11	13
Folding box	0	1	3	6	3
Carton box	0	3	14	11	8
Hinged box	9	52	47	29	8
Hinged tin	0	1	9	16	12
Bundle	0	16	1	0	0
Slide lid box	0	10	1	0	0
Shell/hull & slide box	0	5	7	18	38
All other	4	4	3	1	5
**Pack size**					
Items per pack, mean (SD)	26 (20)	21 (49)	35 (21)	30 (25)	17 (14)
Products with pack size <20 items (%)	3	39	27	41	85
Products with pack size <10 items (%)	1	14	11	19	35

* Only for those used in at least 5% of any group. IQR: interquartile range. SD: standard deviation.

### Cigarillo subtype classification

**Figure 1:**
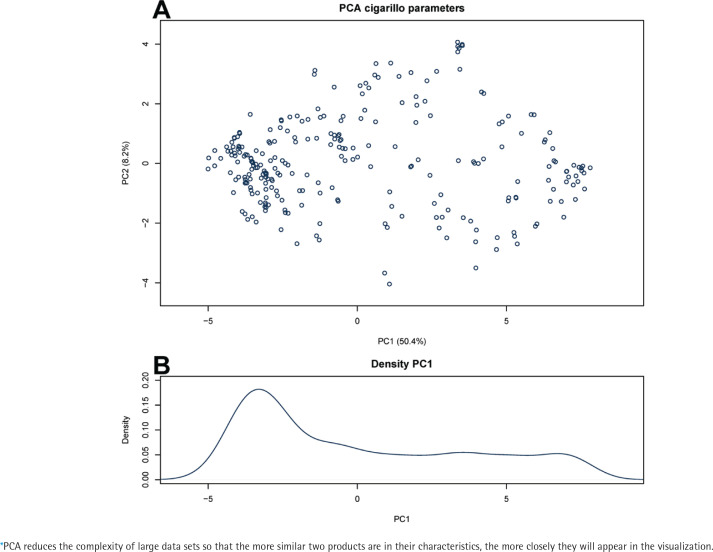
Principal component analysis on cigarillo product parameters: A) PCA* visualization for 285 cigarillos notified as active on the Dutch market on 1 June 2022. Axis units are in arbitrary units. Percentage values on the axes indicate the percentage of total variation between cigarillos that is explained by each axis; B) data distribution of the products’ first principal component coordinate

Data for the 72 product parameters were visualized by PCA, which is shown in Figure 1 A. More than half of the variation (50.4%) between products is represented by principal component 1 (PC1), i.e. the horizontal axis, with PC2 only explaining 8.2%. It can be seen from Figure 1 A that the products are more concentrated on the left side of the plot, with a more gradual distribution towards the right. This was corroborated by the data density plot for PC1, shown in Figure 1 B. This indicates that the overall set of cigarillos notified for the Netherlands consists of groups with different product characteristics.

**Figure 2:**
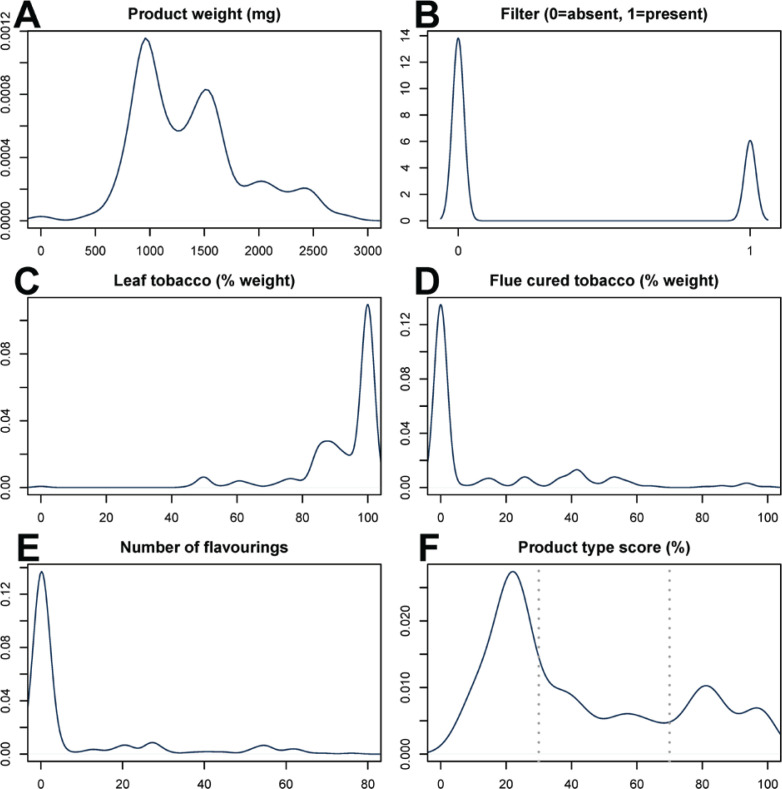
Data distribution densities: A-E) distribution for the five key parameters among cigarillos. Values on the horizontal axis are as indicated in the figure header. Values on the vertical axis are indicated in arbitrary units based on the units on the horizontal axis and the smoothing bandwidth; F) distribution for the logistic regression-based product-type score. Dotted lines indicate cutoff values between cigar-like, intermediate-type and cigarette-like cigarillos

To determine if there are indeed two (or more) groups that can be distinguished among cigarillos and to what extent such a grouping corresponds to a presumed cigar-like/cigarette-like distinction, we determined which of the parameters had such characteristics: 16 parameters showed a bimodal (or multimodal) distribution for cigarillos, in other words indicated originating from two (or more) groups; 36 parameters discriminated between cigars and cigarettes with an ROC AUC of at least 0.9; and 16 parameters met both criteria (Supplementary file Table 1). We noted that there was some redundancy among this set of parameters, by which we mean that two or more parameters essentially provide the same information to distinguish between products groups (for example the percentage of Virginia tobacco and the percentage of flue-cured tobacco) and one of these parameters would be sufficient for a prediction model. After redundancy was removed (see Methods for details), five key parameters remained that divide cigarillos in two or more groups as well as discriminate cigarettes from cigars, these included: product weight, presence of a filter, the percentage of leaf tobacco in the product (as opposed to, for example, cut stems), the percentage of flue-cured tobacco in the product, and the number of flavorings added (Figures 2 A-E and Supplementary file Table 1).

By means of logistic regression, we determined a score for each cigarillo that ranged from 0 to 100% for fully cigar-like to fully cigarette-like parameter values, respectively. The distribution of these values is shown in Figure 2 F. Overall, we found that 140 cigarillos were cigar-like, 74 intermediate-type, and 71 cigarette-like.

### Cigarillo subtype comparison

We compared the three subtypes of cigarillos with each other as well as with cigarettes and cigars. These results are shown in Table 1. Among the five key parameters, the product weight and leaf tobacco percentage had high values for cigars, followed by cigar-like, intermediate-type, cigarette-like cigarillos and low values for cigarettes. The values for the filter presence, flue-cured tobacco percentage and number of flavorings showed approximately the reverse order, having high values for cigarettes and cigarette-like cigarillos and low values for cigar-like cigarillos and cigars. Overall, cigarette-like cigarillos were very similar to cigarettes regarding their product weight (median 998 g and 838 mg, respectively) and filter presence (92% and 98%, respectively) (Table 1). Notably, the average number of flavorings was higher in cigarette-like cigarillos (n=43) than in cigarettes (n=16).

Regarding the package type, the majority of cigars are packaged in a ‘hinged box’ (52%) and the prevalence of this package type shows a decrease going from cigar-like (47%) to cigarette-like cigarillos (8%) (Table 1). Conversely, the majority of cigarettes are packaged in a ‘flip-top box’ (86%); the prevalence of this package type shows an increase going from cigar-like (1%) to cigarette-like cigarillos (13%). Additionally, cigarette-like cigarillos show a higher prevalence of a ‘shell/hull & slide box’ package (38%) (Table 1). When taking into account that the last are similar in appearance to a ‘flip-top box’^23^, it can be summarized that cigar-like and cigarette-like cigarillo packaging mainly resemble those of cigars and cigarettes, respectively.

The average pack size is comparable between cigarettes and cigars, but larger for cigar-like cigarillos and smaller for cigarette-like cigarillos (Table 1). It may be noted here that individual products can be sold by multiple pack sizes. The prevalence of small pack sizes shows a more pronounced difference between product (sub)types. According to EU-CEG data on product presentation and packaging, 3% of cigarettes had a minimum package size of <20 items – also known as kiddie packs^24^ – compared to 39% of cigars, 27% of cigar-like cigarillos, 41% of intermediate-type cigarillos and 85% of cigarette-like cigarillos. For pack sizes of <10 items, a similar trend is found with 1%, 14%, 11%, 19% and 35%, respectively (Table 1). This shows that cigarette-like cigarillos are frequently offered for sale in small pack sizes and that these small pack sizes seem specific for cigarette-like cigarillos as they are less frequently found for cigar-like and intermediate-type cigarillos.

### Flavorings in cigarillo subtypes

**Figure 3:**
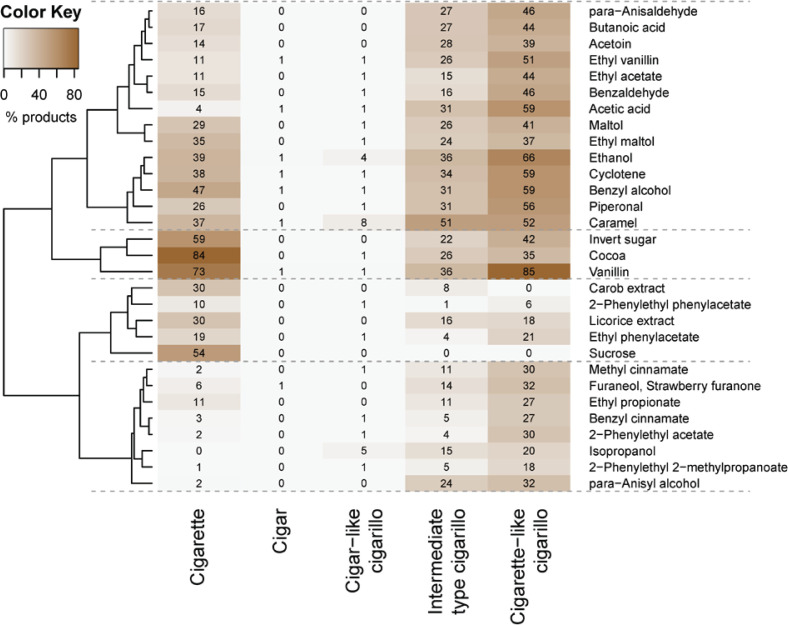
Heatmap for flavorings prevalence per product type. Numerical values and color shades per cell indicate the prevalence of a flavoring in a product (sub)type. The dendrogram on the left clusters flavorings based on similarity of their prevalence profiles. Cluster borders are indicated with dotted lines

A heatmap visualizing the prevalence of flavorings in the various product groups shows four clusters (Figure 3). Flavorings in the top two clusters typically have highest prevalence in cigarette-like cigarillos, somewhat lower prevalence in cigarettes and intermediate-type cigarillos, and low prevalence in cigars and cigar-like cigarillos. These are mostly sweet flavorings, such as cyclotene (which has a ‘caramellic-maple, lovage’ odor and taste), (ethyl) vanillin (‘vanilla’), and maltol (‘sweet, fruity, berry, caramellic’). The third cluster, also comprising sweet flavors, has highest prevalence in cigarettes, followed by cigarette-like and intermediate-type cigarillos. The bottom cluster only shows relevant prevalence in intermediate-type and (especially) cigarette-like cigarillos. This cluster contains flavorings with a sweet-fruity-floral nature, such as furaneol (‘fruity, caramelized pineapple-strawberry’), para-anisyl alcohol (‘sweet, fruity, floral, balsamic anisic-vanilla-creamy-coumarinic like’), and 2-phenylethyl acetate (‘sweet, rose, fruity, honey’).

Taken together, the data in Figure 3 show that many sweet flavorings with high prevalence in cigarettes have even higher prevalence in cigarette-like cigarillos and that the latter group additionally contains several sweet-fruity-floral flavorings. This supports the view that many cigarette-like cigarillos have a characterizing flavor. This is further strengthened by data for two other flavoring substances, which fell short of the criteria for being included in Figure 3 but are of particular interest from a regulatory point of view (being priority additives with enhanced reporting obligations under the TPD)^25^ namely menthol (inhalation facilitating) and diacetyl (respiratory toxicity)^26^. Menthol is found in 0% of cigar-like cigarillos, 4% of intermediate-type and 17% of cigarette-like cigarillos. For diacetyl, these values are 0%, 18% and 25%, respectively.

### Market changes over time

**Figure 4:**
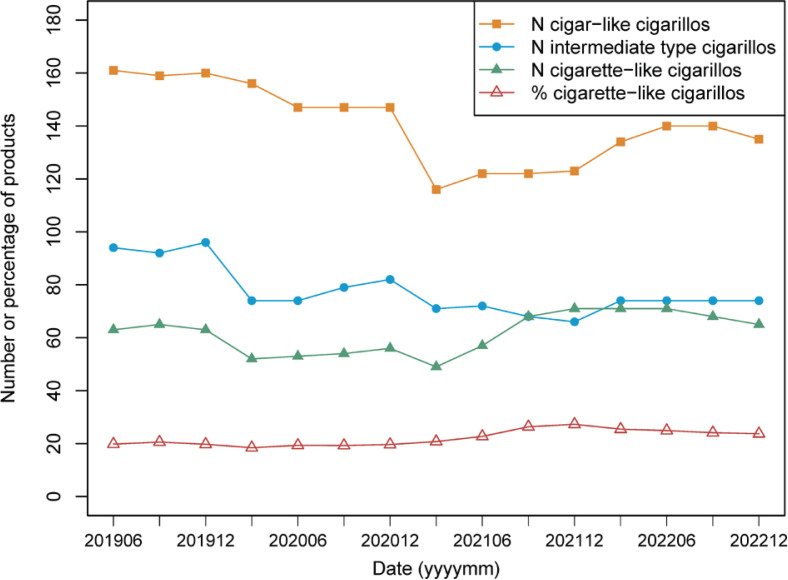
Number or percentage of cigarillo products per type over time

The number of notified products shows a modest but significant decrease over time for cigar-like cigarillos (range: 122–161, Spearman’s rho= -0.71, p=0.003) and intermediate-type cigarillos (range: 66–96, rho = -0.60, p=0.019), whereas the number of cigarette-like cigarillos shows an increase over time that is only just significant (range: 53–71, rho=0.56, p=0.030) (Figure 4). The percentage of cigarette-like cigarillos (as part of the total number for the three subtypes) shows a significant increase over time (range: 18–27%, rho=0.71, p=0.004) (Figure 4). Although all these changes are relatively modest, they point at a shift in the overall market from cigar-like towards cigarette-like cigarillos.

## DISCUSSION

Our analyses confirm our hypothesis that the characteristics of cigarillos range from more cigar-like to more cigarette-like. However, this distinction is not absolute. This was already apparent from the unsupervised PCA, but even with a set of five discriminating product parameters, the resulting product-type score showed an intermediate zone between these two types. This led us to divide cigarillos into three groups, but it must be noted that this classification was done for the purpose of this study and does not imply a qualification or label for individual products. Also, the product-type score does not significantly depend on a single parameter with a single cut-off that might otherwise have served as a convenient threshold for regulation.

The cigarette-like cigarillos comprise almost a quarter of the products notified for the Dutch market. They resemble cigarettes in several product parameters, such as product weight and filter presence, that are easily recognized by consumers. They have a high average number of flavorings, even more than cigarettes. Many, mainly sweet, flavorings with a high prevalence of use in cigarettes have an even higher prevalence in cigarette-like cigarillos. Additionally, several especially sweet-fruity-floral flavorings are found with high prevalence in cigarette-like cigarillos. This indicates that many cigarette-like cigarillos have a characterizing flavor and may serve as alternatives for flavored cigarettes. The package types of cigarette-like cigarillos are more like cigarettes than those of cigars, or even of cigar-like cigarillos. However, especially cigarette-like cigarillos are available in pack sizes of <20 items, which according to Article 14 of the TPD^1^ is the minimal pack size for cigarettes. Hence, cigarette-like cigarillos, despite their cigarette-style packaging, are frequently offered for sale in small pack sizes that would violate EU-regulations for cigarettes. All these characteristics make them attractive products for consumers, including young people. Therefore, this subtype is the most notable from a regulatory point of view, followed by the intermediate-type cigarillo.

The percentage of notified cigarillos that are cigarette-like has increased over the last three years. Although this may suggest an increased popularity among consumers, sales data for individual cigarillo products, as well as consumer studies, would be needed to draw further conclusions on how the different cigarillo types are used among various population groups, such as young people.

For our analysis, we used data as provided by EU-CEG. This database is a valuable resource as it allows collecting a large amount of product data without the need for laboratory studies. It also allows data analyses for products that are no longer active on the market, thus allowing trend analyses such as in Figure 4. However, EU-CEG data alone are not sufficient to conclude on the presence or absence of a characterizing flavor as for example proposed in a report of the USA Food and Drug Administration^27^. A characterizing flavor may depend on a concerted effect of multiple flavorings as well as the tobacco matrix. Although our findings indicate that some cigarette-like cigarillos have a characterizing flavor, they do not allow for statements on individual products, even those labeled with terms such as ‘menthol’ or ‘vanilla’. Definite judgements on this would require sensory analysis by a panel.

The choice for using EU-CEG data also meant that some product characteristics were not available for analysis. These include, for example, the graphic design of the packaging, availability of the brand and the price at point-of-sale, as well as how a characterizing flavor is appreciated by users or even different user groups. Such data can be valuable for understanding the product appeal and use for cigarillo subtypes, as well as the potential impact of legislative changes. However, such data are not as readily available as the EU-CEG data and collecting them would require considerable time. Moreover, the nature of these data (e.g. the packaging design) would not always be suitable for the primarily quantitative approach that we used in this study. Therefore, more research into such other factors is needed.

Our findings touch upon several aspects of tobacco legislation, such as flavor and packaging, and from a policy perspective it is relevant to see how our findings relate to other studies regarding cigarillo users or product characteristics. Given the ban on flavored cigarettes in the EU, especially the substitutability of flavored cigarillos for cigarettes should be a point of attention. Among Dutch adults, the use of cigarillos in 2020 was higher among smokers and highest among participants who indicated a preference for menthol flavored cigarettes. These overrepresentations of smoking characteristics were overall more pronounced than those of personal characteristics^13^. This indicates that flavored cigarillos can act as a substitute for flavored cigarettes. On the other hand, a 2023 US survey among people who smoke menthol cigarettes found that reactions to smoking menthol flavored little cigars or cigarillos (LCC) were mostly unfavorable^28^. Another US study from 2021 found that flavoring was the most popular reason for LCC use among White users while affordability was the most popular among Black/African American users^29^. Although all these studies underline the role of (menthol) flavor in tobacco use, they also point at differences between user groups based on smoking or personal characteristics. A similar nuanced picture emerges regarding packaging and pricing, based on recent studies from the US. Mead-Morse et al.^30^ carried out hypothetical purchase tasks in a sample of LCC smokers and found that lower sensitivity to price changes was correlated with greater use and dependence. A study by Ganz et al.^9^ found that price was rated as more important among Hispanic/Latino and lower income smokers and current cigarette and blunt smokers, hence policies such as a flavor ban or minimum pack sizes could impact sub-populations of cigarillo users differently ^9^. Finally, Jensen et al.^31^ found that regulating LCC packaging could decrease their consumption without increasing cigarette consumption, but effects may differ across jurisdictions depending on prevalence of use. It can be remarked here that in the US, 2.9% of adults report current LCC use^32^, whereas in the EU, 0.5% of adults reported current cigarillo use^33^. Filippidis et al.^33^ compared cigarillo use between EU member states and found considerable variation in cigarillo ever use between countries. Additionally, male sex, living in urban area, and aged >55 years were associated with greater odds of ever cigarillo use. In the same study, it was found that cigarillo prices did not follow the same pattern as cigarette prices between countries. This may be due to differences in national taxation schemes, but also by different marketing strategies of the tobacco industry. Cigarillos may be marketed as luxurious, high-end products in some markets and as affordable alternatives to cigarettes in others^33^.

Taken together, these studies indicate that there is probably no straightforward answer to whether cigarette-like cigarillos should be seen as a type of cigarillo with product characteristics – such as flavor and package size – which makes them attractive for user groups such as young people; or whether they present a flavored cigarette in disguise. Moreover, the impact of policy measures regarding cigarillo product characteristics such as flavor, packaging and pricing may vary between user groups based on their smoking status, personal characteristics and even country of residence. Nevertheless, cigarette-like cigarillos are designed in such a way that their characteristics make them attractive for consumers, including young people. Therefore, it is worth considering to amend current regulations to address this, while keeping in mind how this may affect different user groups.

## CONCLUSIONS

Almost a quarter of the cigarillos notified for the Dutch market have clearly cigarette-like properties but many of these would not meet the regulations on flavor and packaging that apply to cigarettes. In the interest of public health, it can be recommended to address this, either by additional regulations for cigarillos or extending – among others – current cigarette flavor and packaging regulations to include cigarillos.

## Data Availability

The data supporting this research are available from the authors on reasonable request.
