# Supplementary material for: Circumventing cigarette regulation: Product characteristics of cigarette-like cigarillos on the Dutch market
Source: Tob Induc Dis. 2023 Jul 14;21:91. doi: 10.18332/tid/167476 (PMC10347962; doi:10.18332/tid/167476)
Supplement: Supplementary file 1 [file TID-21-91-s1.pdf]

Parameter group	Parameter	Bimodal distribution	AUC cigar - cigarette	Both criteria?	Nonredundant selection key parameters?
General	Weight	strong	0.977	yes	yes
	Length	no	0.912	no	
	Diameter	no	0.967	no	
	TobaccoWeight	weak	0.959	no	
	Filter (true/false)	strong	0.990	yes	yes
	FilterLength	strong	0.991	yes	no, use filter (true/false)
	Number of presentations	weak	0.770	no	
Tobacco composition	TobPart1, Leaf	strong	0.999	yes	yes
	TobPart2, Cut stems	strong	0.975	yes	no, correlated with Virginia and flue-cured
	TobPart3, Reconstituted	weak	0.885	no	
	TobPart4, Expanded	weak	0.902	no	
	TobPart5, Other	no	0.500	no	
	TobType1, Virginia	strong	1.000	yes	no, Virginia is mostly flue-cured, use that as more informative
	TobType2, Burley	weak	0.983	no	
	TobType3, Oriental	weak	0.951	no	
	TobType4, Maryland	weak	0.500	no	
	TobType5, Kentucky	no	0.502	no	
	TobType6, Dark	strong	0.915	yes	no, use Virginia/flue cured instead of Dark/air cured
	TobType7, Other	no	0.599	no	
	TobType8, Unspecified	no	0.718	no	
	TobCure1, Air	strong	0.916	yes	no, use Virginia/flue cured instead of Dark/air cured
	TobCure2, Fire	no	0.502	no	
	TobCure3, Steam	no	0.501	no	
	TobCure4, Sun	weak	0.822	no	
	TobCure5, Flue	strong	0.993	yes	yes
	TobCure6, Other	no	0.802	no	
Number of additives	Total	strong	0.997	yes	no, number of flavour additives more useful
	All except flavour	strong	0.998	yes	no, number of flavour additives more useful
by function	Addictive Enhancer	no	0.500	no	
	Adhesive	weak	0.878	no	
	Binder	no	0.997	no	
	Carrier	no	0.765	no	
	Colour	weak	0.931	no	
	Combustion Modifier	no	0.962	no	
	Casing	no	0.577	no	
	Fibre	no	0.971	no	
	Filler	no	0.997	no	
	Filter Component	no	0.524	no	
	Filtration Material	no	0.923	no	
	Flavour and/or Taste Enhancer	strong	0.901	yes	yes
	Humectant	no	0.962	no	
	pH Modifier	no	0.503	no	
	Plasticiser	no	0.943	no	
	Preservative	no	0.617	no	
	Solvent - Processing Aid	weak	0.819	no	
	Reduced Ignition Propensity Agent	no	0.538	no	
	Sizing Agent	no	0.933	no	
	Smoke Enhancer	no	0.500	no	
	Smoke Colour Modifier	no	0.500	no	
	Smoke Odour Modifier	no	0.500	no	
	Wrapper	no	0.500	no	
	Water-Wetting Agents	no	0.542	no	
	Viscosity Modifier	no	0.562	no	
	Other	no	0.722	no	
by category	tobacco (burnt)	strong	0.950	yes	no, as many of these are flavourings
	tobacco (unburnt)	no	0.502	no	
	paper (burnt)	no	0.999	no	
	side seam adhesive (burnt)	no	0.993	no	
	inks used on cigarette paper (burnt)	no	0.669	no	
	filtration material (unburnt)	no	0.990	no	
	filter overwrap (unburnt)	no	0.985	no	
	filter adhesive (unburnt)	no	0.984	no	
	tipping paper and tipping paper inks (unburnt)	strong	0.988	yes	no, this relates to filter, use that instead
	adhesive (unburnt)	no	0.763	no	
	adhesive (burnt)	no	0.587	no	
	tips (unburnt)	no	0.500	no	
	pouch material (unburnt)	no	0.500	no	
	paper (unburnt)	no	0.501	no	
	other (unburnt)	no	0.500	no	
Additive weight	Total	strong	0.930	yes	no, most additives are not flavourings
	Flavour and/or Taste Enhancer	weak	0.896	no	
	All except flavour	strong	0.926	yes	no, most additives are not flavourings

© 2023 Pennings J.L.A. et al.
